# Supplementary material for: SARS-CoV-2 mRNA vaccines decouple anti-viral immunity from humoral autoimmunity
Source: Nat Commun. 2023 Mar 9;14:1299. doi: 10.1038/s41467-023-36686-8 (PMC9996559; doi:10.1038/s41467-023-36686-8)
Supplement: Supplementary file 1 — Supplementary Information [file 41467_2023_36686_MOESM1_ESM.pdf]

Figure S1:

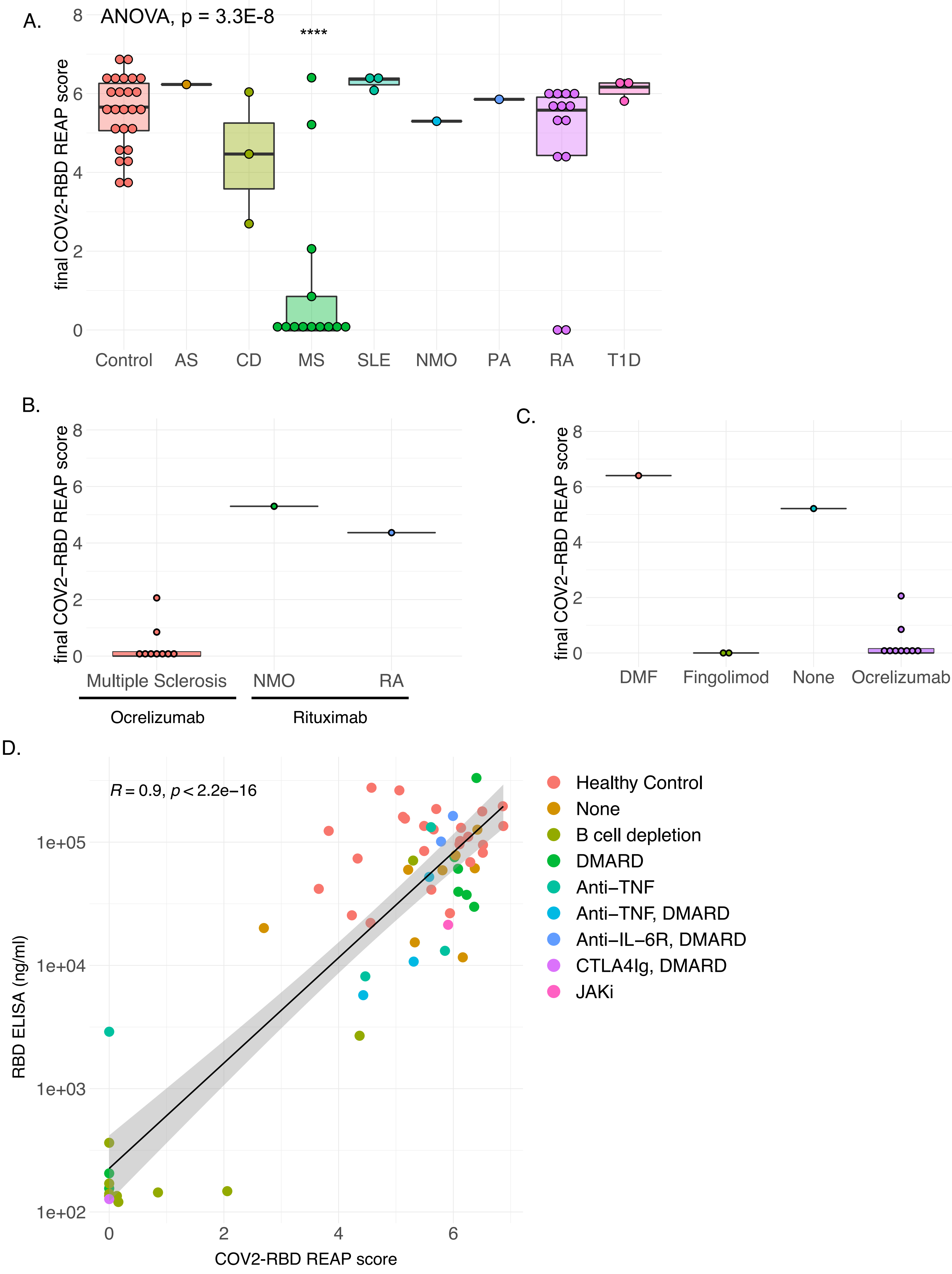
A.
ANOVA, p = 3.3E-8
****
final COV2-RBD REAP score
Control
AS
CD
MS
SLE
NMO
PA
RA
T1D
B.
final COV2−RBD REAP score
Multiple Sclerosis
NMO
RA
Ocrelizumab
Rituximab
C.
final COV2−RBD REAP score
DMF
Fingolimod
None
Ocrelizumab
D.
R = 0.9, p < 2.2e−16
RBD ELISA (ng/ml)
1e+05
1e+04
1e+03
1e+02
COV2-RBD REAP score
Healthy Control
None
B cell depletion
DMARD
Anti−TNF
Anti−TNF, DMARD
Anti−IL−6R, DMARD
CTLA4Ig, DMARD
JAKi

Figure S2:

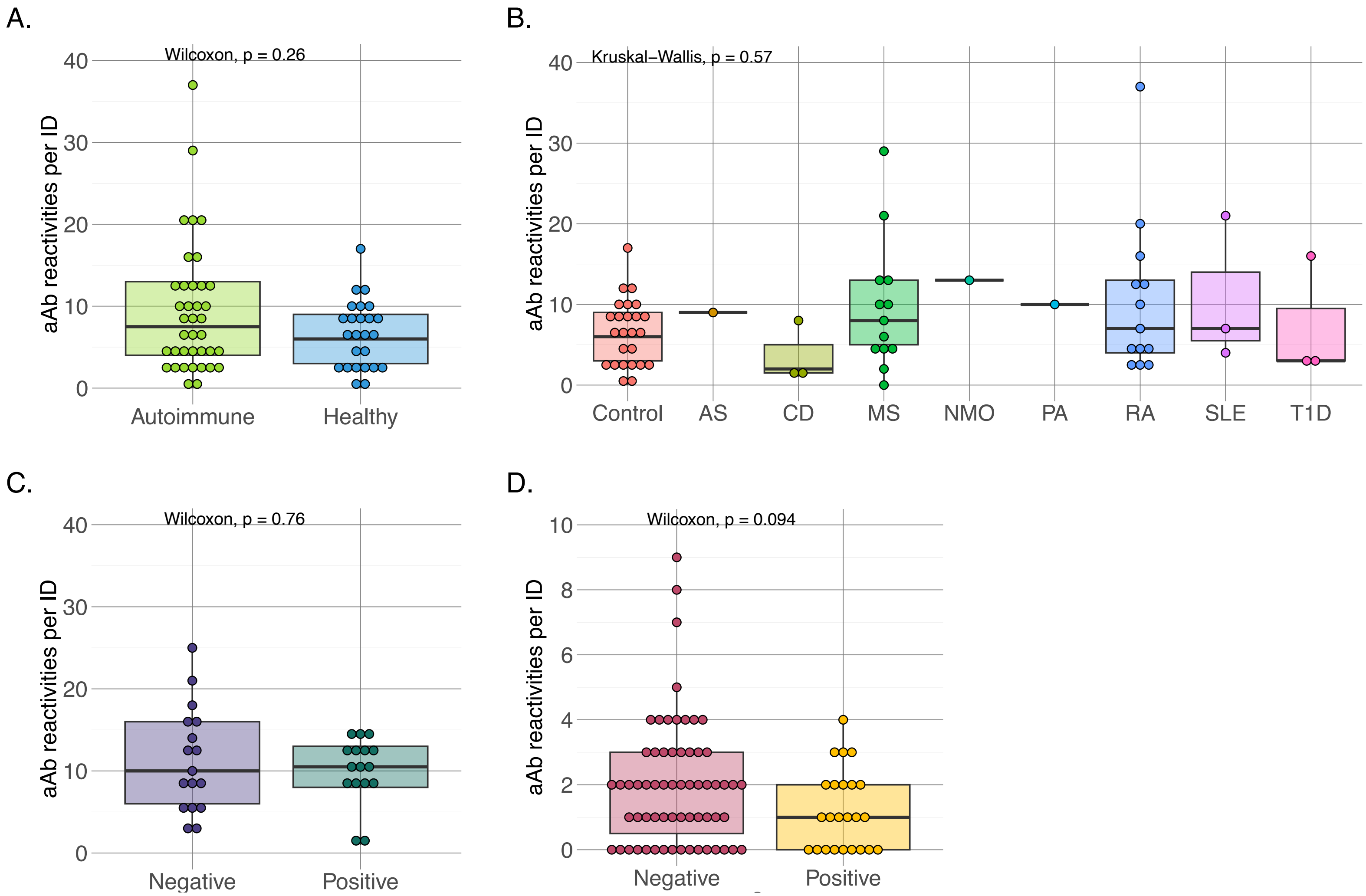
A.
Wilcoxon, p = 0.26
aAb reactivities per ID
0
10
20
30
40
Autoimmune
Healthy
B.
Kruskal–Wallis, p = 0.57
aAb reactivities per ID
Control
AS
CD
MS
NMO
PA
RA
SLE
T1D
C.
Wilcoxon, p = 0.76
aAb reactivities per ID
Negative
Positive
D.
Wilcoxon, p = 0.094
aAb reactivities per ID
0
2
4
6
8
10
Negative
Positive

Figure S3:

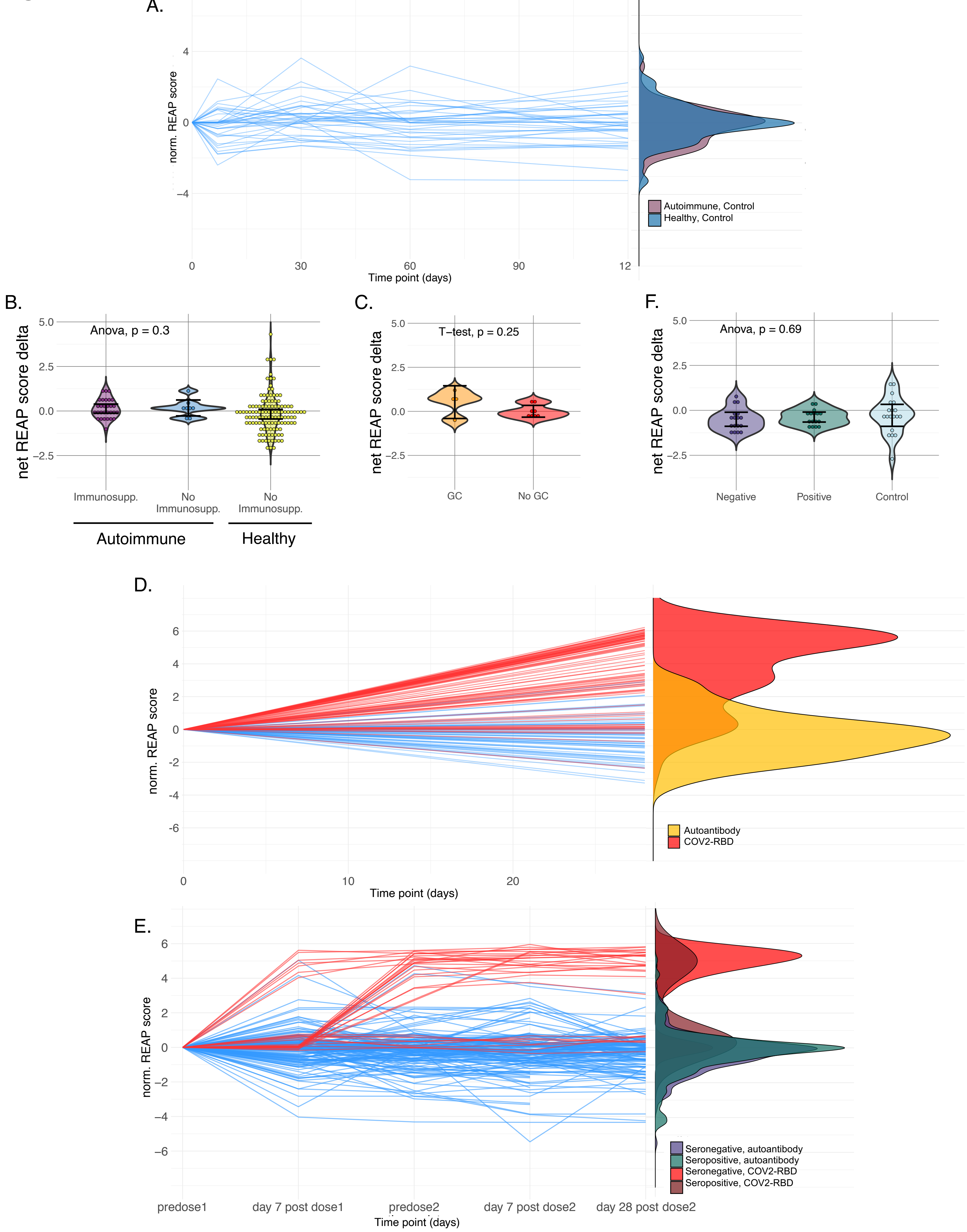

A.
norm. REAP score
4
0
−4
0
30
60
90
12
Time point (days)
Autoimmune, Control
Healthy, Control
B.
net REAP score delta
5.0
2.5
0.0
−2.5
Anova, p = 0.3
Immunosupp.
No Immunosupp.
No Immunosupp.
Autoimmune
Healthy
C.
net REAP score delta
T−test, p = 0.25
GC
No GC
F.
net REAP score delta
Anova, p = 0.69
Negative
Positive
Control
D.
norm. REAP score
6
4
2
0
-2
-4
-6
0
10
20
Time point (days)
Autoantibody
COV2-RBD
E.
norm. REAP score
predose1
day 7 post dose1
predose2
day 7 post dose2
day 28 post dose2
Time point (days)
Seronegative, autoantibody
Seropositive, autoantibody
Seronegative, COV2-RBD
Seropositive, COV2-RBD

Figure S4:

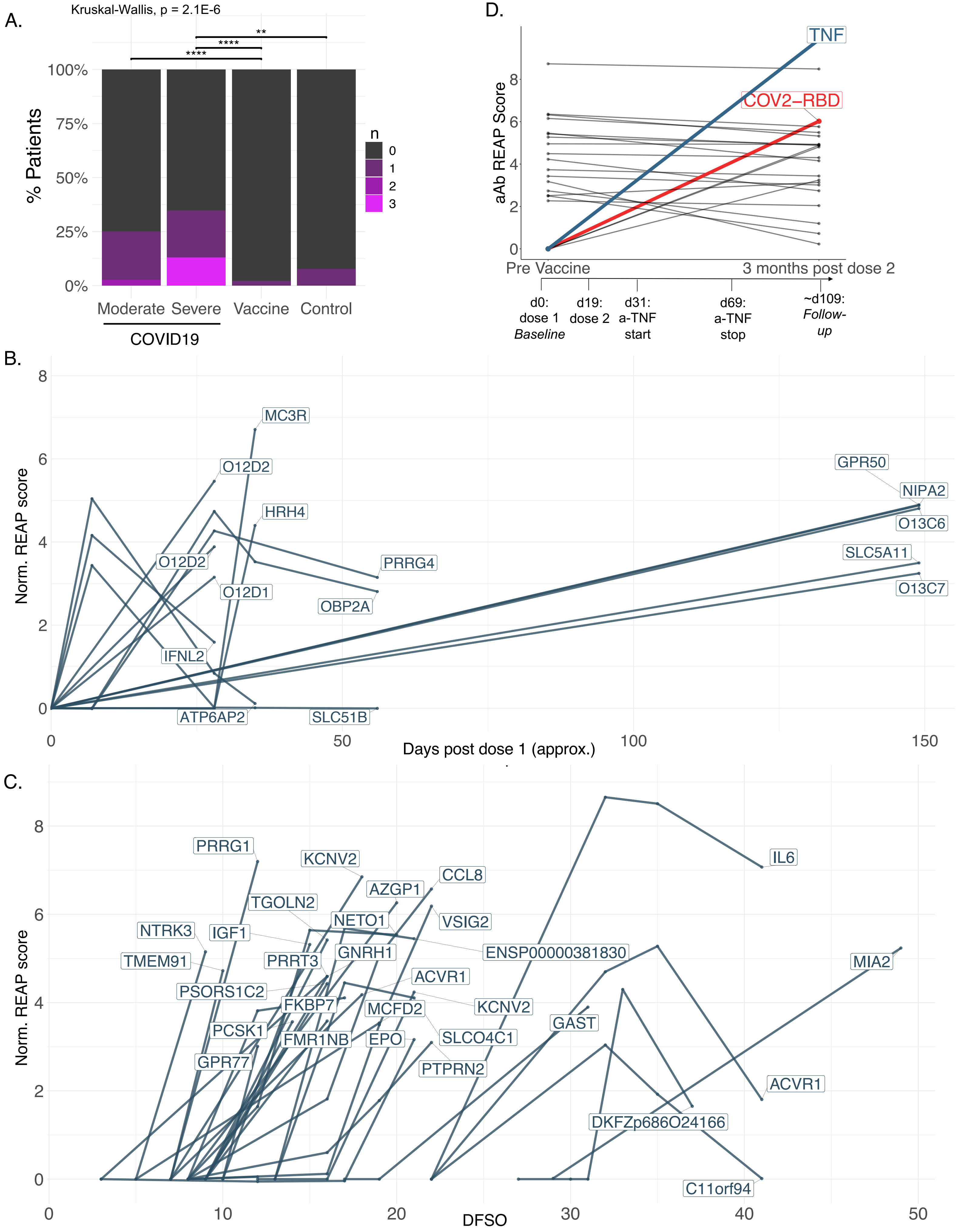
A.
Kruskal-Wallis, p = 2.1E-6
**
****
****
100%
75%
50%
25%
0%
% Patients
n
0
1
2
3
Moderate
Severe
Vaccine
Control
COVID19
D.
TNF
COV2–RBD
aAb REAP Score
8
6
4
2
0
Pre Vaccine
3 months post dose 2
d0: dose 1 Baseline
d19: dose 2
d31: a-TNF start
d69: a-TNF stop
~d109: Follow-up
B.
Norm. REAP score
MC3R
O12D2
HRH4
O12D2
PRRG4
O12D1
OBP2A
IFNL2
ATP6AP2
SLC51B
GPR50
NIPA2
O13C6
SLC5A11
O13C7
Days post dose 1 (approx.)
0
50
100
150
C.
Norm. REAP score
PRRG1
KCNV2
CCL8
AZGP1
TGOLN2
NETO1
VSIG2
NTRK3
IGF1
ENSP00000381830
TMEM91
PRRT3
GNRH1
ACVR1
PSORS1C2
FKBP7
MCFD2
KCNV2
GAST
PCSK1
FMR1NB
EPO
SLCO4C1
GPR77
PTPRN2
IL6
MIA2
ACVR1
DKFZp686O24166
C11orf94
DFSO
0
10
20
30
40
50

Figure S5:

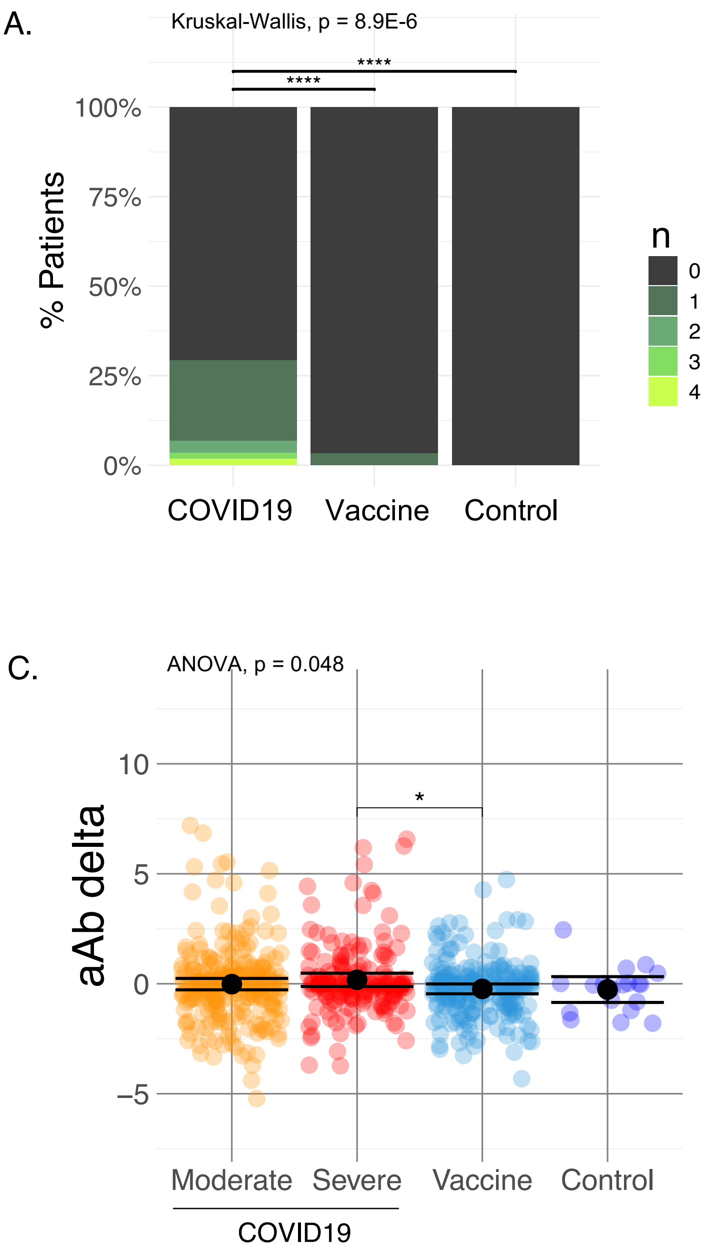
A.
Kruskal-Wallis, p = 8.9E-6
****
****
% Patients
100%
75%
50%
25%
0%
COVID19
Vaccine
Control
n
0
1
2
3
4
B.
Kruskal-Wallis, p = 1.6E-4
***
****
% Patients
100%
75%
50%
25%
0%
COVID19
Vaccine
Control
n
0
1
2
3
C.
ANOVA, p = 0.048
*
aAb delta
10
5
0
−5
Moderate
Severe
Vaccine
Control
COVID19

Figure S6:

A.

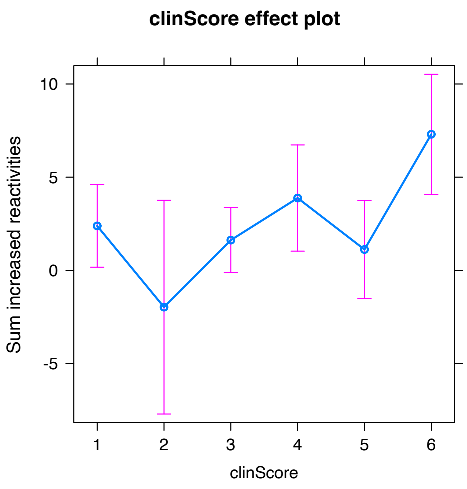

B.

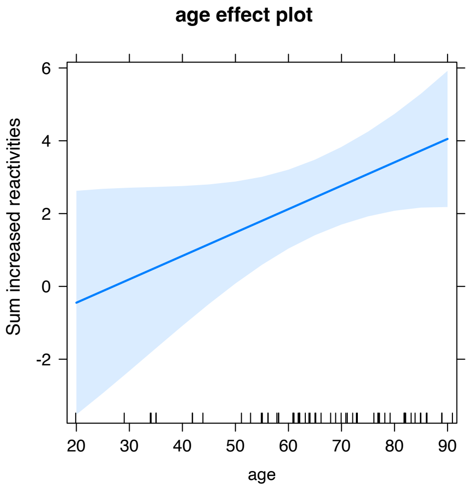

C.

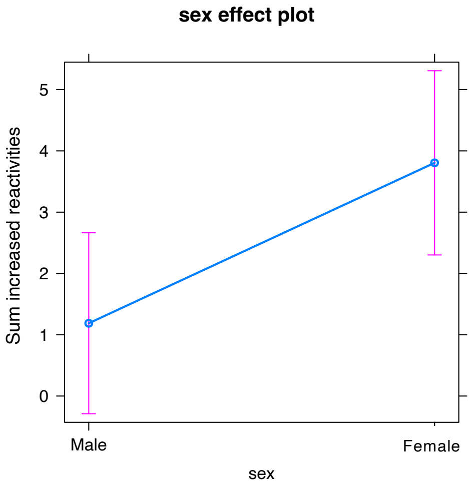

D.

	Estimate	Std. error	t value	Pr(>|t|)	significance
(Intercept)	-3.11163	2.34345	-1.328	0.1902	ns
clinScore2	-4.35789	3.0766	-1.416	0.1627	ns
clinScore3	-0.76068	1.41596	-0.537	0.5935	ns
clinScore4	1.49745	1.79106	0.836	0.407	ns
clinScore5	-1.26444	1.72066	-0.735	0.4658	ns
clinScore6	4.91887	1.94	2.536	0.0143	*
age	0.06427	0.03175	2.024	0.0482	*
sex-Female	2.61715	1.07506	2.434	0.0185	*

Residual standard error: 3.927 on 51 degrees of freedom
Multiple R-squared: 0.2777, Adjusted R-squared: 0.1785
F-statistic: 2.8 on 7 and 51 DF, p-value: 0.01518

E.

	Estimate	Std. error	t value	Pr(>|t|)	significance
(Intercept)	-2.406729	1.637986	-1.469	0.147	ns
Vaccine - Pfizer	0.318215	1.044253	0.305	0.762	ns
Age	0.042078	0.027969	1.504	0.138	ns
sex-Male	1.251725	0.948354	1.32	0.192	ns
time span	0.011509	0.007246	1.588	0.118	ns
Disease category - Healthy	-0.199715	0.787323	-0.254	0.801	ns

Residual standard error: 2.501 on 57 degrees of freedom
Multiple R-squared: 0.08516, Adjusted R-squared: 0.004912
F-statistic: 1.061 on 5 and 57 DF, p-value: 0.3914

Figure S7:

A.

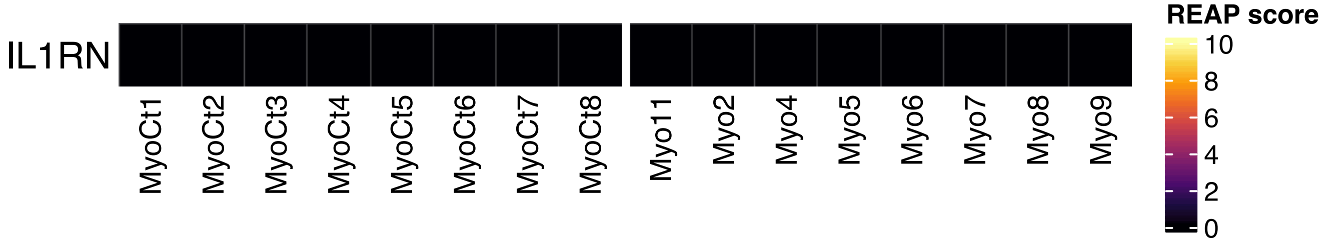
IL1RN
MyoCt1
MyoCt2
MyoCt3
MyoCt4
MyoCt5
MyoCt6
MyoCt7
MyoCt8
Myo11
Myo2
Myo4
Myo5
Myo6
Myo7
Myo8
Myo9
REAP score
10
8
6
4
2
0

Supplementary table 1: Yale HCW Cohort Demographics

		Vaccine		Sex		Serostatus	
	Age (years)	Moderna	Pfizer	Female	Male	Positive	Negative
% Total	44.4	78.8	21.2	81.8	18.2	48.5	51.5
% Seronegative	43.6	58.8	41.2	82.4	17.6		
% Seropositive	45.3	100	0	81.3	18.8		

ID	Sex	SARS-CoV2 prior infection	Vaccine
HCW.1.0008	F	Negative	Pfizer
HCW.1.0009	F	Negative	Pfizer
HCW.1.0023	F	Positive	Moderna
HCW.1.0029	F	Positive	Moderna
HCW.1.0057	F	Negative	Moderna
HCW.1.0074	F	Negative	Moderna
HCW.1.0090	F	Positive	Moderna
HCW.1.0158	F	Positive	Moderna
HCW.1.0205	F	Negative	Pfizer
HCW.1.0222	F	Negative	Pfizer
HCW.1.0223	M	Positive	Moderna
HCW.1.0236	F	Negative	Moderna
HCW.1.0278	F	Positive	Moderna
HCW.1.0288	F	Negative	Moderna
HCW.1.0297	M	Positive	Moderna
HCW.1.0318	M	Negative	Moderna
HCW.1.0324	F	Positive	Moderna
HCW.1.0347	F	Positive	Moderna
HCW.1.0356	F	Positive	Moderna
HCW.1.0365	F	Positive	Moderna
HCW.1.0422	F	Negative	Pfizer
HCW.1.0436	F	Positive	Moderna
HCW.1.0445	F	Positive	Moderna
HCW.1.0447	F	Negative	Moderna
HCW.1.0453	F	Positive	Moderna
HCW.1.0467	M	Negative	Moderna
HCW.1.0582	F	Negative	Moderna
HCW.1.0596	F	Negative	Pfizer
HCW.1.0605	F	Positive	Moderna
HCW.1.0607	F	Negative	Moderna
HCW.1.0614	F	Negative	Moderna
HCW.1.0633	M	Positive	Moderna
HCW.1.0636	M	Negative	Pfizer

Supplementary table 2: Benaroya Cohort Demographics

		Vaccine		Sex		Pre-dose time point		Post-dose time point		Disease	
	Age (years)	Moderna	Pfizer	Female	Male	day of dose 1	<1 month prior to dose 1	2 weeks post dose 2	3 months post dose 2	Healthy	Autoimmune
Total cohort (%)	44	84.4	15.6	81	19	34.9	65.1	39.7	60.3	39.7	60.3
Healthy control (%)	37	88	12	60	40	32	68	32	68		
Autoimmune Disease (%)	48	83.8	18.4	94.7	5.3	36.8	63.2	44.7	55.3		

ID	Race	Sex	Hispanic /Latino	Disease	Prior SARS-CoV2 infection	Pre-vaccine time point	Post-vaccine time point	Vaccine	Medication Categories	Glucocorticoids
1	White, Caucasian	F	no	Ankylosing Spondylitis	naïve	up to 1 month prior to dose 1	3 months post dose 2	Moderna	DMARD	No
2	White, Caucasian	M	no	Control	Negative	day of dose 1	2 weeks post dose 2	Pfizer	None	NA
3	White, Caucasian	F	no	Control	naïve	up to 1 month prior to dose 1	3 months post dose 2	Moderna	None	NA
4	White, Caucasian	M	no	Control	naïve	up to 1 month prior to dose 1	3 months post dose 2	Moderna	None	NA
5	Pacific Islander, White, Caucasian	F	yes	Control	naïve	up to 1 month prior to dose 1	3 months post dose 2	Moderna	None	NA
6	Asian	F	no	Control	naïve	up to 1 month prior to dose 1	3 months post dose 2	Moderna	None	NA
7	White, Caucasian	F	no	Control	naïve	up to 1 month prior to dose 1	3 months post dose 2	Moderna	None	NA
8	White, Caucasian	M	no	Control	naïve	up to 1 month prior to dose 1	3 months post dose 2	Moderna	None	NA
9	Decline	F	yes	Control	naïve	up to 1 month prior to dose 1	3 months post dose 2	Moderna	None	NA
10	White, Caucasian	M	no	Control	naïve	up to 1 month prior to dose 1	3 months post dose 2	Moderna	None	NA
11	White, Caucasian	F	no	Control	Negative	day of dose 1	2 weeks post dose 2	Pfizer	None	NA
12	White, Caucasian	M	no	Control	Negative	day of dose 1	2 weeks post dose 2	Moderna	None	NA
13	Asian	F	no	Control	naïve	up to 1 month prior to dose 1	3 months post dose 2	Moderna	None	NA
14	White, Caucasian	F	no	Control	naïve	up to 1 month prior to dose 1	3 months post dose 2	Moderna	None	NA
15	White, Caucasian	F	no	Control	naïve	up to 1 month prior to dose 1	3 months post dose 2	Moderna	None	NA
16	White, Caucasian	M	no	Control	naïve	up to 1 month prior to dose 1	3 months post dose 2	Moderna	None	NA
17	Asian	F	no	Control	naïve	up to 1 month prior to dose 1	3 months post dose 2	Moderna	None	NA
18	White, Caucasian	M	no	Control	Negative	day of dose 1	2 weeks post dose 2	Moderna	None	NA
19	White, Caucasian	M	no	Control	naïve	up to 1 month prior to dose 1	3 months post dose 2	Moderna	None	NA
20	White, Caucasian	M	no	Control	Negative	day of dose 1	2 weeks post dose 2	Moderna	None	NA
21	Asian	F	no	Control	Negative	day of dose 1	2 weeks post dose 2	Pfizer	None	NA
22	Asian, White, Caucasian	F	no	Control	naïve	up to 1 month prior to dose 1	3 months post dose 2	Moderna	None	NA
23	White, Caucasian	F	decline	Control	Negative	day of dose 1	2 weeks post dose 2	Moderna	None	NA
25	Asian	F	no	Control	naïve	up to 1 month prior to dose 1	3 months post dose 2	Moderna	None	NA
26	White, Caucasian	M	no	Control	Negative	day of dose 1	2 weeks post dose 2	Moderna	None	NA
27	White, Caucasian	F	no	Control	naïve	up to 1 month prior to dose 1	3 months post dose 2	Moderna	None	NA
28	White, Caucasian	F	no	Crohns Disease	naïve	up to 1 month prior to dose 1	3 months post dose 2	Moderna	None	No
29	White, Caucasian	F	no	Crohns Disease	naïve	up to 1 month prior to dose 1	3 months post dose 2	Moderna	None	No
30	White, Caucasian	F	no	Crohns Disease	naïve	up to 1 month prior to dose 1	3 months post dose 2	Moderna	Anti-TNF	No
31	White, Caucasian	F	no	Multiple Sclerosis	Negative	day of dose 1	2 weeks post dose 2	Moderna	DMARD	No
32	White, Caucasian	F	no	Multiple Sclerosis	Negative	day of dose 1	2 weeks post dose 2	Moderna	B cell depletion	No
33	White, Caucasian	M	no	Multiple Sclerosis	Negative	day of dose 1	2 weeks post dose 2	Moderna	B cell depletion	No
34	White, Caucasian	M	no	Multiple Sclerosis	Negative	day of dose 1	2 weeks post dose 2	Moderna	B cell depletion	No
35	White, Caucasian	F	no	Multiple Sclerosis	naïve	up to 1 month prior to dose 1	3 months post dose 2	Moderna	B cell depletion	No
36	White, Caucasian	F	no	Multiple Sclerosis	naïve	up to 1 month prior to dose 1	3 months post dose 2	Moderna	None	No
37	White, Caucasian	F	no	Multiple Sclerosis	Negative	up to 1 month prior to dose 1	2 weeks post dose 2	Moderna	B cell depletion	No
38	White, Caucasian	F	no	Multiple Sclerosis	Negative	day of dose 1	2 weeks post dose 2	Pfizer	B cell depletion	No
39	White, Caucasian	F	no	Multiple Sclerosis	naïve	up to 1 month prior to dose 1	3 months post dose 2	Pfizer	B cell depletion	No
40	White, Caucasian	F	no	Multiple Sclerosis	Negative	day of dose 1	2 weeks post dose 2	Pfizer	B cell depletion	No
41	White, Caucasian	F	no	Multiple Sclerosis	Negative	day of dose 1	2 weeks post dose 2	Pfizer	DMARD	No
42	White, Caucasian	F	no	Multiple Sclerosis	Negative	day of dose 1	2 weeks post dose 2	Pfizer	DMARD	No
43	White, Caucasian	F	no	Multiple Sclerosis	Negative	up to 1 month prior to dose 1	2 weeks post dose 2	Moderna	B cell depletion	No
44	White, Caucasian	F	no	Narcolepsy, SLE	naïve	up to 1 month prior to dose 1	3 months post dose 2	Moderna	DMARD	No
45	Asian, White, Caucasian	F	no	NMO	Negative	day of dose 1	2 weeks post dose 2	Moderna	B cell depletion	No
46	White, Caucasian	F	no	Psoriatic Arthritis	naïve	up to 1 month prior to dose 1	3 months post dose 2	Moderna	Anti-TNF	No
47	White, Caucasian	F	no	RA	Negative	day of dose 1	2 weeks post dose 2	Moderna	DMARD, Anti IL-6R	No
48	White, Caucasian	F	no	RA	naïve	up to 1 month prior to dose 1	3 months post dose 2	Moderna	Anti-TNF, DMARD	Yes, stopped during vax series
49	Asian	F	no	RA	Negative	day of dose 1	2 weeks post dose 2	Pfizer	DMARD, Anti IL-6R	No
50	White, Caucasian	F	no	RA	naïve	up to 1 month prior to dose 1	3 months post dose 2	Moderna	DMARD	No
51	Asian	F	no	RA	naïve	up to 1 month prior to dose 1	3 months post dose 2	Moderna	Anti-TNF	Yes, stopped prior to vax series
52	White, Caucasian	F	no	RA	Negative	day of dose 1	2 weeks post dose 2	Moderna	Anti-TNF, DMARD	Yes
53	White, Caucasian	F	no	RA	naïve	up to 1 month prior to dose 1	3 months post dose 2	Moderna	Anti-TNF	No
54	White, Caucasian	F	no	RA	Negative	up to 1 month prior to dose 1	2 weeks post dose 2	Moderna	CTLA4Ig, DMARD	No
55	White, Caucasian	F	no	RA	naïve	up to 1 month prior to dose 1	3 months post dose 2	Moderna	JAKi	No
56	White, Caucasian	F	no	RA	Negative	day of dose 1	2 weeks post dose 2	Pfizer	B cell depletion	No
57	Asian	F	no	RA	Negative	day of dose 1	2 weeks post dose 2	Moderna	Anti-TNF	No
58	White, Caucasian	F	no	RA	naïve	up to 1 month prior to dose 1	3 months post dose 2	Moderna	DMARD	Yes
59	White, Caucasian	F	no	RA - JIA	naïve	up to 1 month prior to dose 1	3 months post dose 2	Moderna	None	No
60	White, Caucasian	F	yes	SLE	naïve	up to 1 month prior to dose 1	3 months post dose 2	Moderna	None	No
61	Black, African American	F	no	SLE	naïve	up to 1 month prior to dose 1	3 months post dose 2	Moderna	DMARD	No
62	White, Caucasian	F	no	Type 1 Diabetes	naïve	up to 1 month prior to dose 1	3 months post dose 2	Moderna	None	No
63	White, Caucasian	F	no	Type 1 Diabetes	naïve	up to 1 month prior to dose 1	3 months post dose 2	Moderna	None	No
64	White, Caucasian	F	no	Type 1 Diabetes	naïve	up to 1 month prior to dose 1	3 months post dose 2	Moderna	None	No

Supplementary table 3: CoronaVac mRNA Booster Cohort Demographics

	Age range (years)	BMI	Sex		Prior COVID19		Days since last SARS-CoV2 infection	Days since second dose CoronaVac
			Female	Male	Positive	Negative		
% Total	37.5 - 41.5	27.6	68.7	31.3	27.6	72.4		112
% no prior COVID19	38.5 - 42.5	27.8	83	17			338	107

ID	Sex	Prior SARS-CoV2 infection	Number of prior SARS-CoV2 infections	Days since last SARS-CoV2 infection	Days elapsed between CoronaVac second dose and mRNA Booster
1	M	No	0	0	132
2	F	No	0	0	51
3	M	No	0	0	132
5	F	No	0	0	132
6	F	No	0	0	132
7	F	No	0	0	132
8	F	No	0	0	132
9	F	No	0	0	133
11	F	No	0	0	132
13	M	No	0	0	132
16	F	No	0	0	122
18	F	No	0	0	66
19	F	No	0	0	35
20	M	No	0	0	112
21	F	No	0	0	131
22	F	No	0	0	36
23	F	No	0	0	133
24	F	No	0	0	133
26	F	No	0	0	136
27	M	No	0	0	133
28	F	No	0	0	132
29	F	No	0	0	133
30	F	No	0	0	133
31	M	No	0	0	122
32	M	No	0	0	133
33	F	No	0	0	59
34	M	No	0	0	46
35	F	No	0	0	133
36	F	No	0	0	132
37	F	No	0	0	133
39	F	No	0	0	133
40	F	No	0	0	55
41	F	No	0	0	55
42	M	No	0	0	109
43	F	No	0	0	136
44	F	No	0	0	126
45	F	No	0	0	136
46	F	No	0	0	136
48	M	No	0	0	61
50	F	No	0	0	124
51	F	No	0	0	111
52	F	No	0	0	67
53	M	No	0	0	80
54	F	No	0	0	127
55	M	No	0	0	137
56	M	No	0	0	48
57	F	No	0	0	72
58	F	No	0	0	127
59	M	No	0	0	137
60	F	No	0	0	41
61	F	No	0	0	70
62	M	No	0	0	125
63	F	No	0	0	137
64	F	No	0	0	127
65	M	No	0	0	138
66	F	No	0	0	139
67	M	No	0	0	65
69	M	No	0	0	53
71	F	No	0	0	140
72	F	No	0	0	43
73	M	No	0	0	144
74	F	No	0	0	70
75	F	No	0	0	68
76	F	Yes	1	400	128
77	M	Yes	1	491	132
78	M	Yes	1	465	132
79	F	Yes	1	430	132
80	M	Yes	1	491	121
82	F	Yes	1	203	133
83	F	Yes	1	193	133
85	F	Yes	1	462	133
86	M	Yes	1	217	133
87	F	Yes	2	95	133
88	F	Yes	1	462	133
89	F	Yes	1	196	136
90	F	Yes	1	373	136
91	M	Yes	1	465	136
92	M	Yes	1	390	136
93	F	Yes	1	223	76
94	F	Yes	1	410	126
95	M	Yes	1	221	126
96	F	Yes	1	391	127
97	F	Yes	1	380	127
98	F	Yes	1	244	127
99	F	Yes	1	526	35
100	M	Yes	1	163	105
101	F	Yes	1	221	127

Supplementary table 4: Benaroya Longitudinal Control Cohort

		Sex		Disease	
	Age	Female	Male	Healthy	Autoimmune
% Total	29.2	46.1	53.9	27.6	72.4
% Healthy	28.1	57.1	42.9		
% Autoimmune	30.3	33.3	66.6		

ID	Sex	Race	Hispanic/Latino	Disease	Time points (days)
1	M	White, Caucasian	yes	Healthy	0, 7, 30, 60, 90, 120
2	M	White, Caucasian	no	Type 1 Diabetes	0, 7, 30, 60, 90, 120
3	M	White, Caucasian	yes	Type 1 Diabetes	0, 7, 30, 60, 90, 120
4	M	White, Caucasian	no	Type 1 Diabetes	0, 7, 30, 60, 90, 120
5	M	White, Caucasian	no	Type 1 Diabetes	0, 7, 30, 60, 90, 120
6	F	White, Caucasian	no	Type 1 Diabetes	0, 7, 30, 60, 90, 120
7	M	White, Caucasian	no	Type 1 Diabetes	0, 7, 30, 60, 90, 120
8	F	White, Caucasian	no	Healthy	0, 7, 30, 60, 90, 120
9	F	White, Caucasian	no	Type 1 Diabetes	0, 7, 30, 60, 90, 120
10	M	White, Caucasian	no	Healthy	0, 7, 30, 60, 90, 120
11	F	White, Caucasian	no	Healthy	0, 7, 30, 60, 90, 120
12	F	White, Caucasian	no	Type 1 Diabetes	0, 7, 30, 60, 90, 120
13	M	White, Caucasian	no	Healthy	0, 7, 30, 60, 90, 120
14	M	White, Caucasian	no	Type 1 Diabetes	0, 7, 30, 60, 90, 120
15	F	White, Caucasian	no	Healthy	0, 7, 30, 60, 90, 120
16	M	White, Caucasian	no	Healthy	0, 7, 30, 60, 90, 120
17	M	Asian	no	Healthy	0, 7, 30, 60, 90, 120
18	F	White, Caucasian	no	Healthy	0, 7, 30, 60, 90, 120
19	M	White, Caucasian	no	Type 1 Diabetes	0, 7, 30, 60, 90, 120
20	M	White, Caucasian	no	Healthy	0, 7, 30, 60, 90, 120
21	F	White, Caucasian	no	Healthy	0, 7, 30, 60, 90, 120
22	F	White, Caucasian	no	Type 1 Diabetes	0, 7, 30, 60, 90, 120
23	F	White, Caucasian	no	Healthy	0, 7, 30, 60, 90, 120
24	M	White, Caucasian	no	Type 1 Diabetes	0, 7, 30, 60, 90, 120
25	F	White, Caucasian	no	Healthy	0, 7, 30, 60, 90, 120
26	F	White, Caucasian	no	Healthy	0, 7, 30, 60, 90, 120

Supplementary table 5: COVID-19 Cohort Demographics

		Sex		Severity	
	Age (years)	Male	Female	Moderate	Severe
Total (%)	65.6	49.2	50.8	61.0	39.0
Moderate (%)	66.5	55.6	44.4		
Severe (%)	64.3	39.1	60.9		

ID	sex	time points (DFSO)	severity	clinical score
INP.1.0019	M	5, 9	moderate	3
INP.1.0022	M	7, 17	moderate	3
INP.1.0024	M	9, 12	moderate	3
INP.1.0028	M	5, 14, 18, 22	severe	5
INP.1.0029	M	12, 16, 21	severe	6
INP.1.0043	M	9, 14, 22, 25, 28	severe	5
INP.1.0045	M	8, 16, 19, 22	severe	4
INP.1.0055	M	3, 16	moderate	3
INP.1.0061	M	9, 14	severe	6
INP.1.0066	M	12, 16	severe	4
INP.1.0078	M	14, 20	severe	4
INP.1.0079	M	8, 16	severe	5
INP.1.0085	M	5, 19	moderate	1
INP.1.0088	M	4, 9	severe	4
INP.1.0091	M	9, 13, 17	severe	6
INP.1.0097	M	9, 13, 25	moderate	3
INP.1.0105	M	15, 22, 25	moderate	1
INP.1.0109	M	8, 12, 15, 20	moderate	3
INP.1.0113	M	13, 18, 24	moderate	1
INP.1.0122	M	3, 7	moderate	1
INP.1.0143	M	7, 10, 15	moderate	1
INP.1.0160	M	10, 13	moderate	3
INP.1.0211	M	38, 45	moderate	1
INP.1.0241	M	11, 14	moderate	3
INP.1.0247	M	7, 15	moderate	3
INP.1.0251	M	17, 19, 31	severe	5
INP.1.0256	M	5, 7	severe	4
INP.1.0257	M	9, 11	severe	5
INP.1.0296	M	24, 27, 31	severe	4
INP.1.0303	M	11, 15	moderate	3
INP.1.0006	F	12, 16	severe	5
INP.1.0007	F	10, 12	moderate	1
INP.1.0010	F	7, 18	moderate	1
INP.1.0013	F	22, 26	moderate	3
INP.1.0023	F	8, 20	severe	5
INP.1.0025	F	9, 13	moderate	2
INP.1.0027	F	3, 12,17, 21	moderate	1
INP.1.0035	F	7, 11	moderate	3
INP.1.0047	F	9, 16	severe	6
INP.1.0053	F	8, 12	moderate	3
INP.1.0054	F	22, 32, 41	severe	6
INP.1.0059	F	12, 16	severe	6
INP.1.0060	F	13, 17, 21	moderate	3
INP.1.0063	F	8, 12, 27, 29, 49	moderate	3
INP.1.0069	F	8, 12, 17	severe	4
INP.1.0074	F	4, 8	moderate	1
INP.1.0095	F	7, 12	moderate	1
INP.1.0110	F	4, 8	moderate	2
INP.1.0132	F	11, 13	moderate	3
INP.1.0135	F	10, 15	moderate	3
INP.1.0207	F	18, 21	moderate	3
INP.1.0244	F	12, 16	moderate	1
INP.1.0246	F	10, 17	moderate	1
INP.1.0259	F	11, 15	moderate	3
INP.1.0269	F	7, 10	moderate	3
INP.1.0292	F	4, 6	moderate	3
INP.1.0294	F	13, 17	severe	5
INP.1.0297	F	27, 30, 31, 33, 37	severe	4
INP.1.0304	F	3, 7	severe	5

Supplementary table 6: Myocarditis Cases and Controls Demographics

ID	Group	Sex	Vaccine	Dose # after which Sx appeared	Sx onset (days post vaccine)	Nucleocapsid IgG	SARS-CoV-2 RNA, NP
MY02	Myocarditis	M	Pfizer	Second	3	Non-reactive	Negative
MY04	Myocarditis	M	Pfizer	Second	2	Non-reactive	Negative
MY05	Myocarditis	M	Pfizer	Second	3	Non-reactive	Negative
MY06	Myocarditis	M	Pfizer	Second	3	Non-reactive	Negative
MY07	Myocarditis	F	Pfizer	Second	3	Non-reactive	Negative
MY08	Myocarditis	M	Pfizer	Second	3	Non-reactive	Negative
MY09	Myocarditis	M	Pfizer	Second	2	Non-reactive	Negative
MY011	Myocarditis	M	Pfizer	Second	3	Non-reactive	Negative

Average age: 16.12 years

ID	Group	Sex
MyoCt1	Myocarditis Control	M
MyoCt2	Myocarditis Control	M
MyoCt3	Myocarditis Control	F
MyoCt4	Myocarditis Control	M
MyoCt5	Myocarditis Control	M
MyoCt6	Myocarditis Control	M
MyoCt7	Myocarditis Control	M
MyoCt8	Myocarditis Control	M

Average age: 16.25 years

Figure S1:

A, CoV-2-RBD REAP score of Benaroya cohort individuals at the final time point, stratified by autoimmune disease diagnosis. p = 3.3E-8, by one-way ANOVA, with post hoc testing performed using Dunnett's test to compare the mean of every column with healthy control (MS vs Control: p = 2.7E-9). AS = Ankylosing Spondylitis; CD = Crohn's Disease; MS = Multiple Sclerosis; SLE = Systemic Lupus Erythematosus; NMO = Neuromyelitis Optica; PA = Psoriatic Arthritis; RA = Rheumatoid Arthritis; T1D = Type 1 Diabetes. Boxplot colored box depicts 25th to 75th percentile of the data, with the middle line representing the median, upper and lower whiskers represent max and min value within 1.5x $75^{th}/25^{th}$ interquartile range, respectively. N= healthy: 25; autoimmune: 38. **B,** final CoV-2-RBD REAP score of individuals on B cell depletion therapy, stratified by autoimmune diagnosis. Boxplot colored box depicts 25th to 75th percentile of the data, with the middle line representing the median. N = MS: 9; NMO: 1; RA: 1. **C,** final CoV-2-RBD REAP score of MS patients, stratified by medication. Boxplot colored box depicts 25th to 75th percentile of the data, with the middle line representing the median. Each dot represents a single individual. N = DMF: 1; Fingolimod: 2; None: 1; Ocrelizumab: 9. **D,** CoV-2-RBD ELISA (ng/ml) versus CoV-2-RBD REAP score stratified by medication category. Linear regression depicts the relationship between COV2-RBD REAP score and S1 RBD ELISA with 95% C.I. shaded. Each dot represents a single individual. N = healthy: 25; autoimmune: 38. (R = 0.9, p <2.2E-16). ****P < 0.0001, ***P < 0.001 **P < 0.01 and *P < 0.05.

Figure S2:

A, B, Number of preexisting autoantibody reactivities per individual in the BRI cohort, stratified by disease category (A) or autoimmune disease diagnosis (B) (all antigens). p = 0.26 by two-sided Wilcoxon rank-sum test and p = 0.57 by Kruskal-Wallis, respectively. Boxplot colored box depicts 25th to 75th percentile of the data, with the middle line representing the median, upper/lower whiskers represent max/min value within 1.5x $75^{th}/25^{th}$ interquartile range, respectively. Probable drug antibodies (a-TNF, a-IL6R) excluded. AS = Ankylosing Spondylitis; CD = Crohn's Disease; MS = Multiple Sclerosis; SLE = Systemic Lupus Erythematosus; NMO = Neuromyelitis Optica; PA =

Psoriatic Arthritis; RA = Rheumatoid Arthritis; T1D = Type 1 Diabetes. N = Healthy: 25, Autoimmune: 38. **C, D,** Number of preexisting autoantibody reactivities per individual in the Yale HCW cohort (C) and CoronaVac Booster cohort (D) (all antigens). p = 0.76, 0.094 by two-sided Wilcoxon rank-sum test, respectively. Boxplot colored box depicts 25th to 75th percentile of the data, with the middle line representing the median, upper/lower whiskers represent max/min value within 1.5x $75^{th}/25^{th}$ interquartile range, respectively. N = Yale HCW: seronegative – 17, seropositive 16; CoronaVac; prior COVID – 24, no prior COVID: 63. Probable drug antibodies (a-TNF, a-IL6R) excluded.

Figure S3:

A, Line plot: autoantibody (blue) and CoV-2 RBD (red) REAP score trajectories of the longitudinal control cohort (all antigens). Each line represents one antibody reactivity normalized to baseline score of 0. **Density plot:** REAP score deltas for reactivities in healthy (blue) and autoimmune individuals (pink). Probable drug antibodies (a-TNF, a-IL6R) excluded. **B**, **C** Average REAP score change for autoantibody reactivities (all antigens) per individual from the first to the final time point in all vaccine cohorts, stratified by autoimmune and immunosuppression status (B) or RA patients on versus off glucocorticoids (GC) (C). p = 0.3 by Anova (B), p = 0.25 by unpaired two-sided t-test (C). Error bars depict the 99% confidence interval. Probable drug antibodies (a-TNF, a-IL6R) excluded. Each dot represents a single individual. Individuals with zero reactivites detected were excluded. N = Autoimmune/Immunosupression: 30; Autoimmune/No immunosuppression: 8 (B) ; Healthy/No immunosuppression: 106 (B); RA no GC = 8 (C); RA on GC = 4 (C). **D, E. Line plot:** autoantibody (blue) and CoV-2 RBD (red) REAP score trajectories of the CoronaVac booster (D) and Yale HCW (E) cohort (Exo201 antigens only). Each line represents one antibody reactivity normalized to baseline score of 0. **Density plot:** REAP score deltas for reactivities identified in CoronaVac cohort (D) and Yale HCW cohort (E) . Probable drug antibodies (a-TNF, a-IL6R) excluded. **F,** Average REAP score change for autoantibody reactivities per individual from pre- to post-vaccination in the Yale HCW cohort and longitudinal control cohort (all antigens). P = 0.69 by one-way ANOVA. Individuals with zero reactivites

detected were excluded. Each dot represents a single individual. N = Seronegative: 17; Seropositive: 16; Control = 19.

Figure S4:

A, Proportion of patients with n new reactivities (Exo201 antigens only). New reactivity defined as a REAP reactivity not present at the first time point that is present at a subsequent time point with a REAP score >3. Significance assessed by Kruskal-wallis (p = 2.1E-6) with Dunnett's test for post-hoc comparisons and correction for multiple comparisons by Holm's method: moderate vs vaccine: p = 4.7E-5; severe vs vaccine: p = 5.1E-7; severe vs control: p = 2.1E-3. Probable drug antibodies (a-TNF, a-IL6R) were excluded. N= severe COVID19: 23; moderate COVID19: 36; Vaccine: 183; Control: 26. **B,C,** REAP score trajectories of all new autoantibodies appearing after vaccination (B) in the BRI, CoronaVac Booster, and Yale HCW cohorts (all antigens) or during acute COVID-19 (C) (Exo201 antigens only). Each line indicates one reactivity, with the antigen name adjacent to each line. For the vaccine cohort, days are approximate. DFSO = days from symptom onset. Probable drug antibodies (a-TNF, a-IL6R) were excluded. **D,** Antibody trajectory of an RA patient who began taking the anti-TNFα biologic adalimumab (CoV-2-RBD – red, autoantibody – grey, therapeutic Ab – blue).

Figure S5:

A, B, Proportion of patients with n increased reactivities (A) and n new reactivities (B) (Exo201 antigens only) after filtering for timepoints less than or equal to 28 days post symptom onset (COVID19), post dose 1 (vaccine), or post study initiation (longitudinal control). New reactivity defined as a REAP reactivity not present at the first time point that is present at a subsequent time point with a REAP score >3. Increased reactivity defined as an increase in REAP score by >3 points at any time point. Significance assessed by Kruskal-wallis (p = 8.9E-6 (A), p = 1.6E-4 (B)) with Dunn's test for post-hoc comparisons and correction for multiple comparisons by Holm's method. COVID19 vs vaccine: p = 2.3E-7 (A), 1.2E-5 (B); COVID19 vs control: p = 8.6E-6 (A), p = 6.1E-4 (B). Probable drug antibodies (a-TNF, a-IL6R) were excluded. N = COVID19: 59; Vaccine: 120; Control: 26. **C**, Autoantibody deltas from the first to the final timepoint under 28

days or less, grouped by cohort and/or disease severity. p = 0.048 by one-way ANOVA, post hoc testing by Tukey HSD test, severe vs vaccine: p = 0.033. Each dot represents one autoantibody (Exo201 antigens only). Probable drug antibodies (a-TNF, a-IL6R) were excluded. N = moderate COVID-19: 273 reactivities, severe COVID-19: 183 reactivities, Vaccine: 220 reactivities, Control: 20 reactivities.

Figure S6:

A,**B,C** Effect plot of clinical severity score (1 to 6, categorical) (A) , age (B), and sex (C) on magnitude of increased autoantibodies in multiple linear regression model. Magnitude of increased autoantibodies calculated by summing the maximum delta REAP score of increased autoreactivities, which were defined as any autoantibody with a score increase of >3. 95% confidence interval shaded. N = 59. **D**, details of the multiple linear model for increased reactivity magnitude in COVID19 patients. **E**, details of the multiple linear regression model for increased reactivity magnitude in Benaroya mRNA vaccine cohort patients. N = 63.

Figure S7:

A, Heatmap depicting the REAP scores for IL1RN in the myocarditis and control cohort.
