# Supplementary material for: SARS-CoV-2 mRNA vaccines decouple anti-viral immunity from humoral autoimmunity
Source: Nat Commun. 2023 Mar 9;14:1299. doi: 10.1038/s41467-023-36686-8 (PMC9996559; doi:10.1038/s41467-023-36686-8)
Supplement: Supplementary file 3 — Reporting Summary [file 41467_2023_36686_MOESM3_ESM.pdf]

nature portfolio

Corresponding author(s): Aaron Ring

Last updated by author(s): 01/31/2023

Reporting Summary

Nature Portfolio wishes to improve the reproducibility of the work that we publish. This form provides structure for consistency and transparency in reporting. For further information on Nature Portfolio policies, see our Editorial Policies and the Editorial Policy Checklist.

Statistics

For all statistical analyses, confirm that the following items are present in the figure legend, table legend, main text, or Methods section.

n/a	Confirmed	
☐	☒	The exact sample size (*n*) for each experimental group/condition, given as a discrete number and unit of measurement
☒	☐	A statement on whether measurements were taken from distinct samples or whether the same sample was measured repeatedly
☐	☒	The statistical test(s) used AND whether they are one- or two-sided<br>*Only common tests should be described solely by name; describe more complex techniques in the Methods section.*
☒	☐	A description of all covariates tested
☐	☒	A description of any assumptions or corrections, such as tests of normality and adjustment for multiple comparisons
☐	☒	A full description of the statistical parameters including central tendency (e.g. means) or other basic estimates (e.g. regression coefficient) AND variation (e.g. standard deviation) or associated estimates of uncertainty (e.g. confidence intervals)
☐	☒	For null hypothesis testing, the test statistic (e.g. *F*, *t*, *r*) with confidence intervals, effect sizes, degrees of freedom and *P* value noted<br>*Give P values as exact values whenever suitable.*
☒	☐	For Bayesian analysis, information on the choice of priors and Markov chain Monte Carlo settings
☒	☐	For hierarchical and complex designs, identification of the appropriate level for tests and full reporting of outcomes
☒	☐	Estimates of effect sizes (e.g. Cohen's *d*, Pearson's *r*), indicating how they were calculated

Our web collection on statistics for biologists contains articles on many of the points above.

Software and code

Policy information about availability of computer code

Data collection	EPIC EHR software (retrospective EMR review and clinical data aggregation) and REDcap (version 9.3.6) (clinical data aggregation)
Data analysis	R (version 4.1.2); tidyverse (version 1.3.0); ggplot2 (version 3.3.5); edgeR (version 3.30.3); tidyr (version 1.1.3); dyplr (version 1.0.7), forcats (version 0.5.1); ggpubr (version 0.4.0); stringr (version 1.4.0); FactoMineR (version 2.4); readr (version 2.0.1); matrixStats (version 0.60.1); ggstance (0.3.5); janitor (2.1.0); ggrepel (0.9.1); ComplexHeatmap (2.8.0)

For manuscripts utilizing custom algorithms or software that are central to the research but not yet described in published literature, software must be made available to editors and reviewers. We strongly encourage code deposition in a community repository (e.g. GitHub). See the Nature Portfolio guidelines for submitting code & software for further information.

Data

Policy information about availability of data

All manuscripts must include a data availability statement. This statement should provide the following information, where applicable:

- Accession codes, unique identifiers, or web links for publicly available datasets
- A description of any restrictions on data availability
- For clinical datasets or third party data, please ensure that the statement adheres to our policy

All data is available from authors upon reasonable request

Field-specific reporting

Please select the one below that is the best fit for your research. If you are not sure, read the appropriate sections before making your selection.

[x] Life sciences [ ] Behavioural & social sciences [ ] Ecological, evolutionary & environmental sciences

For a reference copy of the document with all sections, see nature.com/documents/nr-reporting-summary-flat.pdf

Life sciences study design

All studies must disclose on these points even when the disclosure is negative.

Sample size	No statistical methods were performed to determine the size of the vaccination or acute COVID19 cohorts. For the vaccination cohort, sample size was determined by the number of individuals able to be recruited by the Yale, CoronaVac, or Benaroya study coordinators. For the acute COVID19 cohort, sample size was determined by the number of patients admitted to Yale New Haven Hospital between March 2020 and May 2020 who wished to participate in the study.
Data exclusions	Patients were excluded from the acute COVID19 cohort if they did not have moderate or severe disease or if they only had 1 time point, because this would prohibit the longitudinal analysis required by our study.<br>Patients were excluded from the vaccination cohort if an unclear sample label was detected, which would preclude verification of the origin of the sample. Additionally, one patient was excluded due to failed REAP process and lack of autoantibody data.<br>2 patients were excluded from the Myocarditis cohort: 1 patient received IVIG before their blood sample, which complicates the results of REAP, making them uninterpretable; 1 patient had onset of myocarditis 21 days after the vaccination, which is an atypical time course and thus the cause of the myocarditis (viral vs vaccine) could not be determined.
Replication	REAP analysis was performed in duplicate. Results displayed reflect the average of the two replicates for each sample.<br>ELISA analysis was performed in duplicate and repeated twice.<br>neutralization was performed in a series of 6 dilutions and repeated twice. Results displayed reflect the average of the two repeats for each sample.
Randomization	Randomization is not relevant to the research performed on human samples since those studies were observational in nature. Patients in the Yale vaccination cohort were stratified by prior SARS-CoV-2 infection confirmed by PCR. Patients in the benaroya cohort were stratified by presence of an autoimmune disease. Patients in the acute COVID-19 cohort were stratified by disease severity, based on oxygen levels and ICU requirement. Moderate disease status (Clinical Score 1,2, or 3) was defined as: 1) SARS-CoV-2 infection requiring hospitalization without supplemental oxygen, 2) infection requiring non-invasive supplemental oxygen (<3 L/ min, sufficient to maintain >92% SpO2), 3) infection requiring non-invasive supplemental oxygen (>3 L/min, sufficient to maintain >92% SpO2, or, required >2L supplemental oxygen to maintain SpO2 >92% and had a high-sensitivity C-reactive protein (CRP) >70 and received tocilizimab. Severe disease status (clinical score 4 or 5) was defined as meeting criteria for score 3 while also requiring admission to the YNHH intensive care unit (ICU) and >6L supplemental oxygen to maintain SpO2 >92% (4), or infection requiring invasive mechanical ventilation / extracorporeal membrane oxygenation (ECMO) in addition to gluccocorticoid / vasopressor administration (5). Clinical score 6 was assigned for deceased patients, however in this study, it is included in the severe disease group.
Blinding	At the time of sample acquisition and processing, scientists were unaware of the patients conditions.<br>For the acute COVID-19 cohort and the Benaroya cohort, REAP and ELISA/neutralization studies were performed by the investigator before receiving associated clinical annotations. For the Yale HCW cohort and the myocarditis cohort, the investigator received clinical annotations at the time of REAP/ELISA/neutralization studies, however samples were analyzed in the same manner in a randomized plate layout.

Reporting for specific materials, systems and methods

We require information from authors about some types of materials, experimental systems and methods used in many studies. Here, indicate whether each material, system or method listed is relevant to your study. If you are not sure if a list item applies to your research, read the appropriate section before selecting a response.

Materials & experimental systems

n/a	Involved in the study	
[ ]	[x]	Antibodies
[ ]	[x]	Eukaryotic cell lines
[x]	[ ]	Palaeontology and archaeology
[x]	[ ]	Animals and other organisms
[ ]	[x]	Human research participants
[x]	[ ]	Clinical data
[x]	[ ]	Dual use research of concern

Methods

n/a	Involved in the study	
[x]	[ ]	ChIP-seq
[x]	[ ]	Flow cytometry
[x]	[ ]	MRI-based neuroimaging

Antibodies

Antibodies used	REAP antibodies:<br>acute COVID-19 and Yale HCW cohorts: Biotin anti-human IgG (clone HP6017, 1:100, Biolegend, Discontinued, #409308)<br>Benaroya, CoronaVac, Longitudinal Control, and Myocarditis cohorts: Biotin anti-Human IgG (clone QA19A42, 1:100, Biolegend, 366902)<br>ELISA:<br>HRP anti-Human IgG (1:5000, GenScript, #A00166)<br>Human anti-Spike (AM006415, Active Motif, #91351)<br>Goat anti-human IgG Fc HRP (Sigma Aldrich, #AP113P)<br>Mouse anti-human IL-1RA (Prospec, ant-238)<br>Goat anti-mouse IgG Fc (Thermo Fisher Scientific, #A16088)
Validation	All antibodies used in this study are commercial available or were commercially available at the time of use and since discontinued. All antibodies have thus been validated by the manufacturers and used by other publications. Likewise, we titrated these antibodies according to optimal detection conditions for REAP and ELISA. Further information can be obtained from the vendors' websites.

Eukaryotic cell lines

Policy information about cell lines

Cell line source(s)	TMPRSS2-VeroE6 kidney epithelial cell line was obtained from the ATCC
Authentication	TMPRSS2-VeroE6 was obtained from ATCC, tested and authenticated by morphology, karyotyping, and PCR based approaches.
Mycoplasma contamination	n The cell line tested negative for contamination with mycoplasma.
Commonly misidentified lines<br>(See ICLAC register)	No commonly misidentified cell lines were used in the study

Human research participants

Policy information about studies involving human research participants

Population characteristics	Cohort characteristics:<br>Yale HCW: age (average 44.4), sex (Male 18% / Females 81%), serostatus (48% positive / 51% negative)<br>Benaroya: age (average 44), sex (Male 19% / 81%), Disease (Healthy 40% / Autoimmune 60%)<br>CoronaVac: age (average 37 - 41.5), sex (Male 31.7%/Female 68.3%)<br>Longitudinal control: age (average 29.2), sex (Male 53.9%/Female 46.1%)<br>Acute COVID-19: age (average 65.6), sex (Male 49% / Female 51%), severity (Moderate 61% / Severe 39%)<br>Myocarditis: age (average 15.7), sex (Male 87.5% / Female 12.5%)<br>Full demographic data is included in extended data tables 1-6
Recruitment	Yale HCW vaccination cohort: HCW volunteers from the Yale New Haven Hospital (YNHH) were recruited between November 2020 and January 2021 during SARS-CoV-2 vaccination program. Participants were enrolled during the vaccination program with no self selection. The study goal (characterization of the immune response post vaccination) was explained to the participants. Participants interested in the study , consented with the current study and were recruited. Informed consent was obtained by trained staff and sample collection commenced immediately upon study enrollment.<br>Acute COVID-19 cohort: patients admitted to the YNHH between March 18th to May 18th were recruited to the Yale IMPACT study after testing positive for SARS-CoV-2 by RT-qPCR. Patients were identified through screening of EMR records for potential enrollment with no self-selection. Informed consent was obtained by trained staff and sample collection commenced immediately upon study enrollment. Clinical specimens were obtained approximately every 4 days, where an individuals clinical status permitted.<br>Benaroya cohort: Patients were recruited from Virginia Mason medical system between December 2020 and April 2021. Participants were enrolled during vaccination with no self selection. The study goal was explained to patients, and patients consented to the study. Sample collection began immediately upon recruitment.<br>Coronavac Cohort: The CoronaVac Booster study was approved under the National Bioethics Committee of the Dominican Republic (CONABIOS). The participants received two doses of whole-virion inactivated vaccine CoronaVac, followed by 1 dose of BNT162b2 mRNA vaccine at least four weeks after completion of the CoronaVac course. All participants consented to enroll in this study.<br>Myocarditis cohort: Patients were recruited from YNHH between May and October 2021. Patients presented with symptoms consistent with Myocarditis that was subsequently verified by MRI. Symptom onset for all participants was within 4 days after the second dose of mRNA vaccine. Patients were enrolled if interested iand consented to the study. Sample selection commenced immediately upon study recruitment.
Ethics oversight	Yale HCW, Acute COVID-19, and Myocarditis cohorts: Yale Human Research Protection Program Institutional Review Boards approved this work. Informed consents were obtained from all enrolled participants. IRB protocol numbers:<br>Yale HCW: 2000028924<br>Acute COVID-19: 20000027690<br>Myocarditis: 2000028924 and 1605017838

Benaroya vaccine cohort: Benaroya Research Institute Institutional Review Board approved this work. Informed consents were obtained from all enrolled participants: IRB protocol number: IRB08108
Benaroya control cohort: Benaroya Research Institute Institutional Review Board approved this work. Informed consents were obtained from all enrolled participants: IRB protocol number: IRB10024
Benaroya myocarditis controls: Benaroya Research Institute Institutional Review Board approved this work. Informed consents were obtained from all enrolled participants: IRB protocol number: IRB3041700

Coronavac cohort: Informed consent was obtained from all enrolled participants. The CoronaVac Booster study was approved by the National Bioethics Committee of the Dominican Republic (CONABIOS).

Note that full information on the approval of the study protocol must also be provided in the manuscript.
